# Lasting s-ketamine block of spreading depolarizations in subarachnoid hemorrhage: a retrospective cohort study

**DOI:** 10.1186/s13054-019-2711-3

**Published:** 2019-12-30

**Authors:** Edgar Santos, Arturo Olivares-Rivera, Sebastian Major, Renán Sánchez-Porras, Lorenz Uhlmann, Kevin Kunzmann, Roland Zerelles, Modar Kentar, Vasilis Kola, Adrian Hernández Aguilera, Mildred Gutierrez Herrera, Coline L. Lemale, Johannes Woitzik, Jed A. Hartings, Oliver W. Sakowitz, Andreas W. Unterberg, Jens P. Dreier

**Affiliations:** 1 Neurosurgery Department, Heidelberg University Hospital- Ruprecht-Karls-Universität Heidelberg, Im Neuenheimer Feld 400, 69120 Heidelberg, Germany; 2 Center for Stroke Research Berlin, Charité–Universitätsmedizin Berlin, Freie Universität Berlin, Humboldt-Universität zu Berlin, and Berlin Institute of Health, Berlin, Germany; 3 Department of Neurology, Charité–Universitätsmedizin Berlin, Freie Universität Berlin, Humboldt-Universität zu Berlin, and Berlin Institute of Health, Berlin, Germany; 4 Department of Experimental Neurology, Charité–Universitätsmedizin Berlin, Freie Universität Berlin, Humboldt-Universität zu Berlin, and Berlin Institute of Health, Berlin, Germany; 5 Institute of Medical Biometry and Informatics, Ruprecht-Karls-University Heidelberg, Heidelberg, Germany; 6 Evangelisches Krankenhaus Oldenburg, University of Oldenburg, Oldenburg, Germany; 7 UC Gardner Neuroscience Institute, University of Cincinnati (UC) College of Medicine, Cincinnati, OH USA; 8 Department of Neurosurgery, University of Cincinnati (UC) College of Medicine, Cincinnati, OH USA; 9 Neurosurgery Center Ludwigsburg-Heilbronn, RKH Klinikum Ludwigsburg, Ludwigsburg, Germany; 10 Bernstein Center for Computational Neuroscience Berlin, Berlin, Germany; 11 Einstein Center for Neurosciences Berlin, Berlin, Germany

**Keywords:** Stroke, Subarachnoid hemorrhage, Electrocorticography, Neuromonitoring, Ketamine, Spreading depression

## Abstract

**Objective:**

Spreading depolarizations (SD) are characterized by breakdown of transmembrane ion gradients and excitotoxicity. Experimentally, *N*-methyl-d-aspartate receptor (NMDAR) antagonists block a majority of SDs. In many hospitals, the NMDAR antagonist s-ketamine and the GABA_A_ agonist midazolam represent the current second-line combination treatment to sedate patients with devastating cerebral injuries. A pressing clinical question is whether this option should become first-line in sedation-requiring individuals in whom SDs are detected, yet the s-ketamine dose necessary to adequately inhibit SDs is unknown. Moreover, use-dependent tolerance could be a problem for SD inhibition in the clinic.

**Methods:**

We performed a retrospective cohort study of 66 patients with aneurysmal subarachnoid hemorrhage (aSAH) from a prospectively collected database. Thirty-three of 66 patients received s-ketamine during electrocorticographic neuromonitoring of SDs in neurointensive care. The decision to give s-ketamine was dependent on the need for stronger sedation, so it was expected that patients receiving s-ketamine would have a worse clinical outcome.

**Results:**

S-ketamine application started 4.2 ± 3.5 days after aSAH. The mean dose was 2.8 ± 1.4 mg/kg body weight (BW)/h and thus higher than the dose recommended for sedation. First, patients were divided according to whether they received s-ketamine at any time or not. No significant difference in SD counts was found between groups (negative binomial model using the SD count per patient as outcome variable, *p* = 0.288). This most likely resulted from the fact that 368 SDs had already occurred in the s-ketamine group before s-ketamine was given. However, in patients receiving s-ketamine, we found a significant decrease in SD incidence when s-ketamine was started (Poisson model with a random intercept for patient, coefficient − 1.83 (95% confidence intervals − 2.17; − 1.50), *p* < 0.001; logistic regression model, odds ratio (OR) 0.13 (0.08; 0.19), *p* < 0.001). Thereafter, data was further divided into low-dose (0.1–2.0 mg/kg BW/h) and high-dose (2.1–7.0 mg/kg/h) segments. High-dose s-ketamine resulted in further significant decrease in SD incidence (Poisson model, − 1.10 (− 1.71; − 0.49), *p* < 0.001; logistic regression model, OR 0.33 (0.17; 0.63), *p* < 0.001). There was little evidence of SD tolerance to long-term s-ketamine sedation through 5 days.

**Conclusions:**

These results provide a foundation for a multicenter, neuromonitoring-guided, proof-of-concept trial of ketamine and midazolam as a first-line sedative regime.

## Introduction

Ischemic lesions contribute to poor outcome after aneurysmal subarachnoid hemorrhage (aSAH) [1, 2]. Spreading depolarization (SD) is a key mechanism of lesion development because it initiates the cytotoxic neuronal edema in various gray matter structures [3]. Accordingly, SDs after aSAH occur abundantly [4–6]. Terminal SD was recently recorded in patients who either died from circulatory arrest [7] or suffered brain death despite sustained circulatory function [8, 9] or developed neuroimaging-proven early or delayed ischemic strokes after aSAH in the recording area [10–13]. In the context of lesion progression, terminal SD was typically preceded by a temporal cluster of increasingly prolonged SDs that could start up to hours earlier, suggesting a spiraling trend toward increasing risk of injury [10]. By contrast, isolated SD in eloquent and metabolically intact tissue is the pathophysiological correlate of the harmless migraine aura [14, 15]. SD induces tone alterations in resistance vessels, causing either transient hyperemia (normal hemodynamic response) in healthy tissue or severe hypoperfusion (inverse hemodynamic response = spreading ischemia) in tissue at risk for injury [16–18]. Experimentally, spreading ischemia could be the sole cause of widespread cortical infarcts [19]. In patients, the full continuum from spreading hyperemia to ischemia has been observed in aSAH patients [5], and spreading ischemia was associated with neuroimaging-proven lesion progression [10].

*N*-Methyl-d-aspartate receptor (NMDAR) antagonists block SD in metabolically intact tissue [20] where SD is short-lasting and harmless [21]. By contrast, they progressively fail to do so under elevated baseline K^+^ concentration [22] or in increasingly ischemic or hypoxic tissue [23–26] where SDs are progressively longer-lasting and deleterious. However, an important aspect is that SD, which comes from metabolically intact tissue, can invade tissue where it triggers spreading ischemia [10, 18]. Spreading ischemia then transforms metabolically still slightly disturbed tissue into severely disturbed tissue. NMDAR antagonists could thus prevent metabolic collapse, if they block the trigger, SD, that becomes terminal SD via the vicious circle of SD-induced vasoconstriction [27].

The NMDAR antagonist ketamine has been found to be associated with a reduction in SD occurrence in a mixed population of 60 patients with traumatic brain injury (TBI), 31 patients with aSAH, and 24 patients with malignant hemispheric stroke [28, 29]. The first prospective controlled trial of ketamine for SD inhibition recently confirmed this result in 8 patients with TBI and 2 with aSAH [30, 31]. Also, a case of spontaneous intracerebral hemorrhage has been reported in whom a cluster of SDs disappeared in response to ketamine and reappeared after discontinuation of ketamine [32]. In bedside-to-bench translation, results were further replicated in swine studies in which the active enantiomer, S(+) ketamine (s-ketamine), decreased the expansion, amplitude, and speed of SDs at a dosing of 2 mg/kg body weight (BW)/h. However, complete blockade of SDs in adequately perfused tissue was achieved only at a high dose of 4 mg/kg BW/h [33, 34]. This suggested that the assumed neuroprotective effect of s-ketamine essentially starts at a dose that is above the recommended range, yet is nonetheless administered to patients by neurointensivists in individual cases. Another ketamine problem, previously observed in rats, is the use-dependent development of tolerance [35]. To better understand ketamine treatment, here we retrospectively analyzed a cohort of 66 aSAH patients from a prospectively collected database. We investigated (i) the dose-response relationship of s-ketamine to inhibition of SD occurrence, SD expansion, and amplitude, (ii) whether there is evidence of tolerance development, and (iii) whether s-ketamine affects intracranial (ICP) or mean arterial pressure (MAP).

## Materials and methods

### General

Inclusion criteria were (i) age ≥ 18 years, (ii) World Federation of Neurosurgical Societies scale (WFNS) grades I-V and a Glasgow Coma Score ≥ 4, (iii) ruptured saccular aneurysm proven by computed tomography (CT)-angiography or digital subtraction angiography, and (iv) either surgical treatment of the aneurysm via craniotomy or, in coiled patients, burr hole trepanation for placement of a ventricular drain or oxygen sensor, which allows the simultaneous placement of a subdural electrode strip (Wyler, 5-mm diameter; Ad-Tech Medical, Racine, Wisconsin, USA) [5]. Exclusion criteria were bilaterally fixed and dilated pupils or other signs of imminent death, as well as a history of trauma/bleeding ≥ 5 days before admission. Using these pre-specified criteria, we identified 32 patients from a prospectively collected database of the University of Heidelberg Department of Neurosurgery who had been consecutively recruited between September 2004 and March 2014. Nineteen of these had been treated with s-ketamine at some time point during the electrocorticography (ECoG) monitoring time. Additionally, we identified 33 consecutive patients from a prospectively collected database at Charité–Universitätsmedizin Berlin who had been recruited between April 2005 and March 2009 whereas 4 patients were excluded. In addition, one patient who received s-ketamine and was monitored October 2010 was added to this list. In total, 14 patients from Berlin had been treated with s-ketamine.

**Fig. 1:**
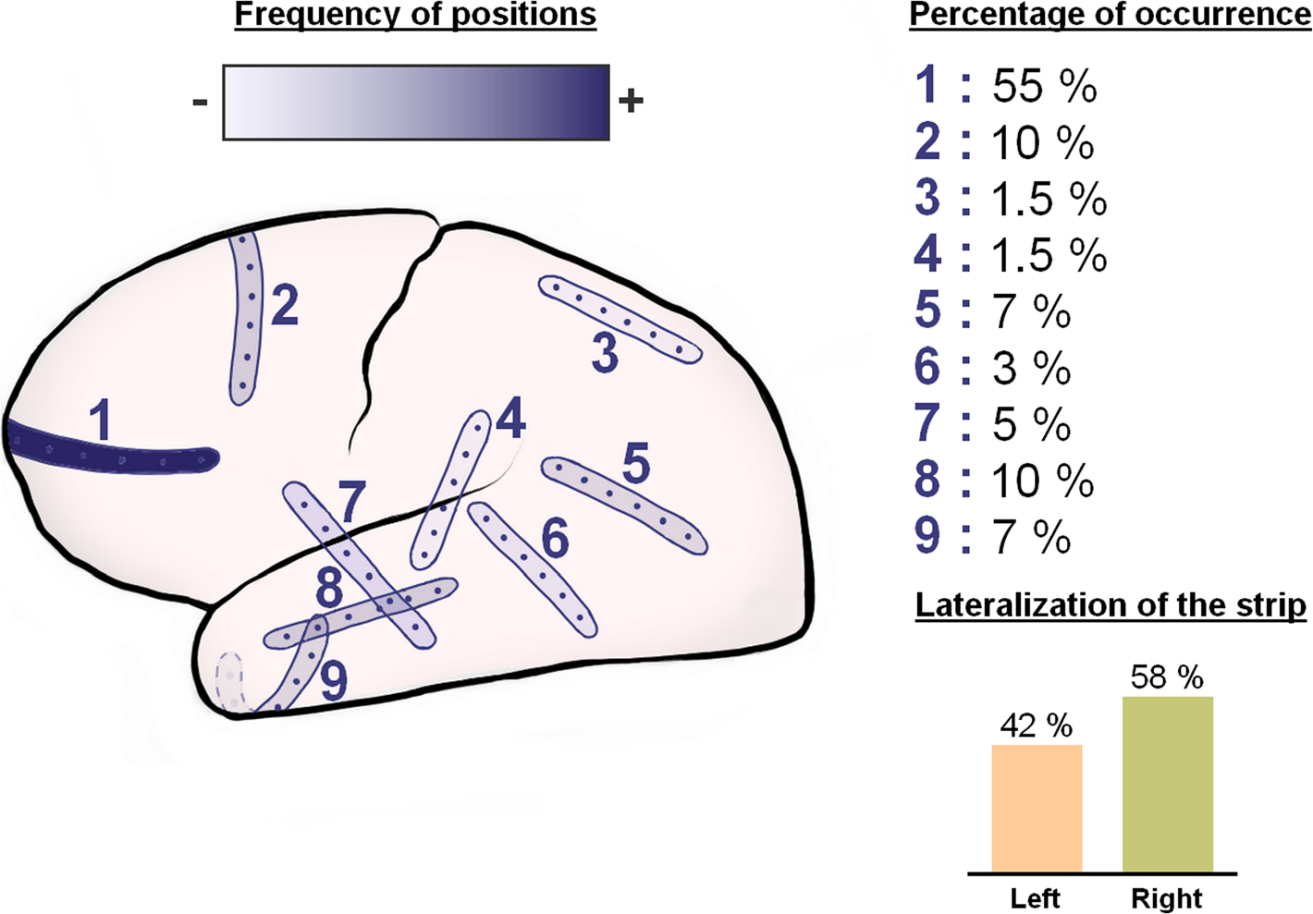
Recording strip density map illustrating the frequency of different locations of the electrode strips in our patient population. The strip locations were taken from the CT topograms

**Fig. 2:**
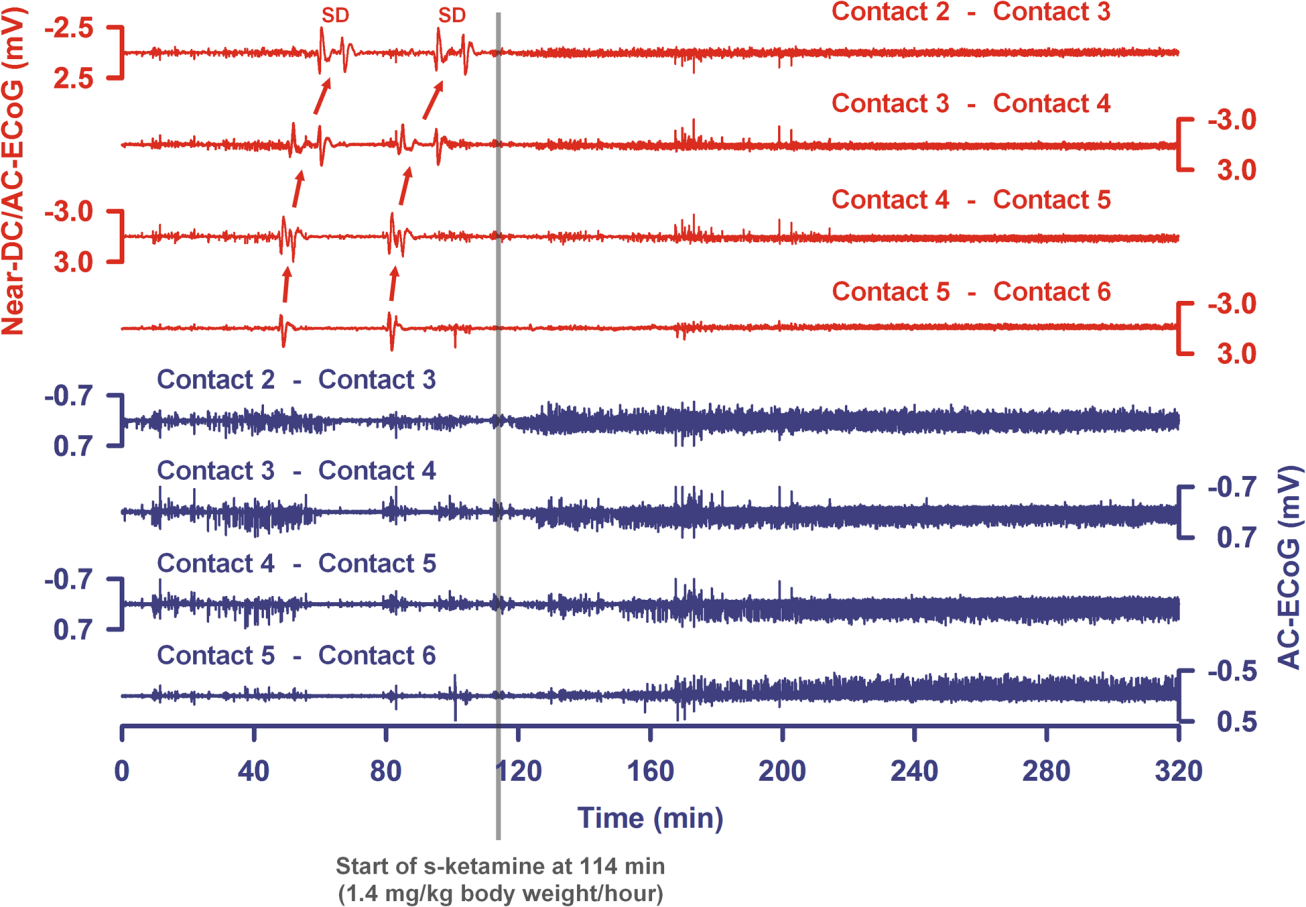
The last two SDs of a cluster are shown from a patient with aSAH, intraventricular, and intracerebral hemorrhage. Top traces show bipolar near-direct current (DC)/alternate current (AC) (frequency band 0.01–45 Hz) recordings from five electrode contacts and bottom traces show high-frequency spontaneous activity from the same electrode contacts (AC, frequency band 0.5–45 Hz). The arrows indicate the spread of the SDs between the electrode contacts. SDs no longer occurred after the intravenous administration of s-ketamine was started. Note that the spontaneous activity then recovered in all channels. The recovery is less pronounced in trace 8

**Fig. 3:**
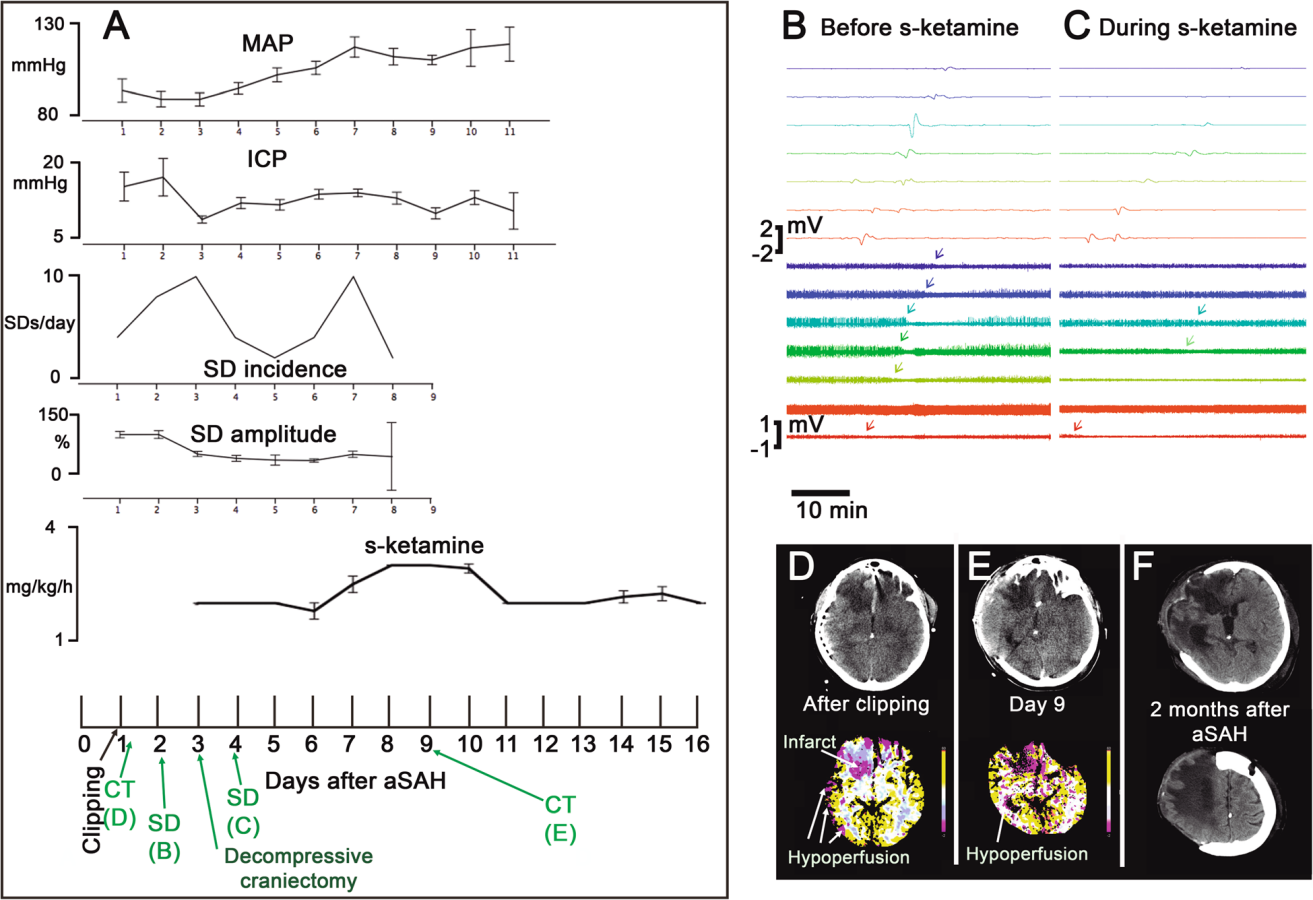
A 55-year-old female with WFNS 4 and modified Fisher grade 4 aSAH [36] due to rupture of an anterior communicating artery aneurysm is presented. On day 1 after the initial hemorrhage, the aneurysm was clipped, extraventricular drainage established, and a subdural electrode strip implanted. From the beginning, the patient showed increased ICP. On day 2, she developed pneumonia. S-ketamine started on day 2.5. On day 3, decompressive hemicraniectomy was performed to control the increased ICP. **a** The plots show MAP, ICP, the incidence of SDs, the amplitudes of the SDs’ near-DC-shifts, and the administered doses of s-ketamine over time. Error bars represent 95% confidence intervals. Note that the ECoG was recorded from day 1 to day 8. On the time axis of the lowest plot, the time points of the CT scans, and ECoG examples shown in **b–f** are indicated in green color. Note that s-ketamine was not sufficient to block the SDs in this case and the incidence of SDs even increased on day 7 despite continued application of s-ketamine. Further dose escalation on day 8 was then associated with another decrease in SD incidence before the electrode strip was removed. **b** SD propagating from electrode contact 8 to 1 before application of s-ketamine. Top traces show monopolar near-direct current (DC)/alternate current (AC) (frequency band 0.01–45 Hz) recordings from eight electrode contacts and bottom traces show high-frequency spontaneous activity from the same electrode contacts (AC, frequency band 0.5–45 Hz). The arrows in the bottom traces mark the start of the SD-induced depression periods. **c** During s-ketamine application, SDs typically propagated from electrode contact 8 to 2. Note that the SD amplitudes are smaller and that the spontaneous activity is now more suppressed than before s-ketamine administration. Accordingly, it is more difficult to see the SD-induced depression periods (arrows in the bottom traces). The smaller amplitudes of SDs and spontaneous activity not necessarily resulted from s-ketamine. It is in fact more likely that they were due to progressive metabolic compromise of the tissue [10]. This would also explain why s-ketamine was insufficient to block all SDs in this case. **d** The CT control post clipping showed infarcts in the right anterior cerebral artery (ACA) territory (frontal base until the head of the caudate nucleus). The CT perfusion maps for cerebral blood flow, cerebral blood volume, mean transit time (MTT), and time to peak revealed hypoperfusion in the right ACA and middle cerebral artery (MCA) territories. As an example, the MTT is shown. **e** On day 9, the patient underwent a control CT after withdrawal of the electrode strip. The CT scan showed the hemicraniectomy and revealed enlargement of the right ACA infarct as well as progressive brain edema with gyral swelling suggesting additional right MCA infarction. **f** Right-sided ACA and MCA infarcts in the late CT control scan 2 months after aSAH

Figure 1 provides an electrode strip density map illustrating in a schematic fashion the typical locations of the subdural electrode strips in our patient population. The near-direct current (DC)/alternate current (AC)-ECoG was recorded in four, five, or seven active channels from either a six-contact or an eight-contact linear electrode array (interelectrode distance 1 cm) with electrode contacts connected in sequential bipolar fashion to a GT205 amplifier (0.01–45 Hz) (ADInstruments, New South Wales, Australia) (Fig. 2). In some patients, contact 1 served as ground. In others, a subdermal platinum needle electrode was additionally placed over the hemisphere ipsilateral to the recording strip. Each contact of the recording strip was then referenced to the subdermal platinum electrode, allowing recordings in monopolar fashion in addition to the bipolar recordings (Fig. 3). Data were sampled at 200 Hz and recorded and analyzed with a Powerlab 16/SP analog/digital converter and Chart-7 software (ADInstruments, New South Wales, Australia). SDs were defined as recommended recently [37]. Accordingly, a cluster of recurrent SDs was defined by the occurrence of at least three SDs occurring within three or fewer consecutive recording hours. Further SDs were counted as clustered SDs when they occurred within less than 1 h after the previous SD.

The anesthesiologic standard procedures were nearly identical at both institutions. Notably, they were not influenced by data from the ECoG monitoring. While the aneurysm was secured, patients were deeply sedated using short-term sedatives and analgesics such as propofol, sevoflurane, remifentanil, and small amounts of sufentanyl. Thereafter, sedation was transiently reduced to create a time window for the assessment of the neurological status. If the patient presented with a new neurological deficit, a CT scan and optional control angiography were performed. Whenever possible, the patient was extubated within the first 6 h after the surgical or endovascular intervention. If this was not possible, the sedation was repeatedly reduced in regular time intervals over the next 1 to 3 days to create further windows for neurological assessment. If predicted from the amount of early brain damage and repeated neurological assessments that prompt recovery would be unlikely, long-term sedation was started using the sedative midazolam and the analgesics sufentanyl and fentanyl. S-ketamine was then usually added for clinical purposes to supplement midazolam, if (i) maximal doses of midazolam and sufentanyl were insufficient to reach adequate analgo-sedation, (ii) ICP continued to peak above the desired level, or (iii) ventilation was difficult. Barbiturates such as thiopental or methohexital were only rarely used and muscle relaxants were avoided. When barbiturates were given, this represented less than 1% of the monitoring time.

For the purpose of the study, patients were divided according to whether or not s-ketamine (Ketanest S®) was administered at any time during the ECoG monitoring. Doses of sedatives, ICP, and MAP were recorded hourly. MAP was recorded at the heart level. Glasgow Coma Score, blood gases, glucose, and electrolytes were documented every 6 h. A thorough neurological examination was performed at least daily. Markers for infection (leucocytes and C-reactive protein (CRP)) were documented daily. Patients received a prophylaxis with oral nimodipine to prevent delayed cerebral ischemia (DCI). Patients with DCI were treated with hyperdynamic therapy as described previously [5]. Clinical outcome was assessed at 6 months according to the extended Glasgow Outcome Scale (eGOS, 1–8) by a neurologist, a neurosurgeon, or a trained study nurse. The large majority of survivors were seen and interviewed in person.

Data was mainly collected and coded by ES and AOR. AOR performed the ECoG analysis blinded to the clinical, pharmacological, and neuroimaging data. As a strategy to avoid errors and bias, the correctness of data coding was proofed by AHA and MGH. An analysis to detect extreme data and unusual data was done by ES. VK performed the analysis of the neuroimaging data blinded to the clinical, pharmacological, and ECoG data. The tables with the raw data were given to the statisticians to perform independently the predefined statistical analysis.

### Statistical analysis

We provide the regression coefficient or the odds ratio for the group variable with 95% confidence intervals (CI) and *p* values. Note that the coefficient estimated in a negative binomial model describes the difference in the logarithm of the expected counts while in a linear model the coefficient can be interpreted as the mean difference between the groups. A *p* value less than 5% was considered as statistically significant. However, all analyses are of explorative nature and have no confirmatory value. Therefore, no adjustment for multiple tests was applied. The analyses were carried out using the software R version 3.3.1 (https://www.r-project.org/) in combination with the packages nlme (https://cran.r-project.org/web/packages/nlme/index.html), lme4 (https://www.jstatsoft.org/article/view/v067i01/0), MASS [38], xtable (https://cran.r-project.org/web/packages/xtable/index.html), and Matrix (https://cran.r-project.org/web/packages/Matrix/index.html).

In a first step, descriptive statistics were separately calculated for patients who received s-ketamine and patients who did not for all outcome variables of interest. For continuous variables, the mean values and standard deviations are provided. For categorical variables, absolute and relative frequencies were analyzed. In a next step, differences in the incidence of SDs and SD characteristics between the two patient groups were examined. Negative binomial models as well as (mixed) Poisson, logistic, or linear models were used as appropriate. When using mixed models, we took account of the clustered structure in the data (several observations per patients were measured) and the resulting correlation structure. The group variable (as a fixed effect) and a random intercept were included in the models as appropriate. In an additional analysis, we did not include the group variable but the indicator, if s-ketamine was given or not (one observation per hour).

## Results

Table 1 gives a demographic summary of the 66 aSAH patients. SDs were detected in 51/66 patients (83%). Thirty-three patients received s-ketamine during the monitoring period and 33 did not. Monitoring data of up to 19 days after aSAH was included in the analysis. S-ketamine administration started 4.2 ± 3.5 days (range 0–16) after aSAH (cf. general protocol of sedation in “Materials and methods”). The mean dose was 2.8 ± 1.4 mg/kg BW/h. S-ketamine was given during a mean of 8.3 ± 4.6 days (range 1–15).

**Table 1:** Summary of the clinical characteristics of the 66 aSAH patients

		No s-ketamine	s-ketamine	*p* value
Number of patients		33	33	
Male		9	15	0.125
Age		55.8 ± 11.7	51.1 ± 8.0	0.064
MAP (mmHg)		95.8 ± 16.3	100.7 ± 8.1	0.125
ICP (mmHg)		13.1 ± 15.0	11.2 ± 4.1	0.500
CPP (mmHg)		83.0 ± 28.2	89.6 ± 8.9	0.219
Pneumonia		16	20	0.323
Urinary tract infection		6	4	0.492
Monitoring days		14.8 ± 4.9	17.0 ± 2.3	0.024
AcoA		9	13	0.649
ACoP		2	1	
BCA		0	1	
ICA		3	3	
MCA		18	15	
PericA		1	0	
Aneurysm diameter		7.5 ± 5.1	7.3 ± 4.4	0.864
WFNS	1	3	2	0.405
	2	7	2	
	3	3	3	
	4	7	11	
	5	13	15	
Modified Fisher Scale	0	0	0	0.198
	1	1	0	
	2	1	0	
	3	10	5	
	4	21	28	
ICH		10	12	0.763
No ICH		23	21	
Infarction	Areal	20	21	0.683
	Lacunar	4	3	
	No	9	9	
eGOS	1	7	8	0.154
	2	0	2	
	3	7	6	
	4	5	8	
	5	2	6	
	6	3	1	
	7	4	1	
	8	4	1	

Neuroimaging was performed following standard care when clinical deterioration was noted. No strict imaging protocol was established for the purpose of the study. Therefore, caution is warranted in the interpretation of the imaging results. CT and/or magnetic resonance imaging (MRI) scans were analyzed to identify focal lesions (infarct or hemorrhage). VK analyzed the neuroimages blinded to the clinical courses and ECoG analyses. An infarct with a diameter ≤ 15 mm was denoted as “lacunar.” An infarct > 15 mm was denoted as “areal.” Hyperintensities in diffusion-weighted imaging (DWI) or hypodensities on CT resulting from ventricular catheters or intraparenchymal hematoma were documented as such. Intracerebral hemorrhages (ICH) appeared hypodense on later CT scans. These hypodensities as well as peri-hematomal hypodensities were denoted as “ICH” and not rated as ischemic lesions. All ICHs occurred during the initial hemorrhage. By contrast, ischemic infarcts could occur early or in a delayed fashion. The table reports whether patients developed an ischemic infarct at any time during the clinical course after the hemorrhage. Welch’s *t*-tests or chi-squared tests were applied as appropriate. *ACoA* anterior communicating artery, *ACoP* posterior communicating artery, *BCA* basilar cerebral artery, *ICA* internal carotid artery, *MCA* middle cerebral artery, *PericA* pericallosal artery, *WNFS* World Federation of Neurosurgical Societies Sheart scale, *eGOS* extended Glasgow Outcome Scale

The anesthesiologic standard procedures, as described in “Materials and methods,” led to a strong selection bias such that patients who received s-ketamine had more severe aSAH (Table 1). Accordingly, (i) 79% of patients receiving s-ketamine but only 61% of patients not receiving s-ketamine showed WFNS 4 or 5 values at admission and (ii) 85% of patients receiving s-ketamine displayed a modified Fisher scale 4 hemorrhage at admission in contrast to only 64% of patients not receiving s-ketamine. Accordingly, the outcome of patients receiving s-ketamine was significantly worse (s-ketamine: eGOS 1–5: *n* = 30, eGOS 6–8: *n* = 3; no s-ketamine: eGOS 1–5: *n* = 21, eGOS 6–8: *n* = 11, Fisher exact test, *p* = 0.017).

### S-ketamine effect on SDs on the patient level

**Fig. 4:**
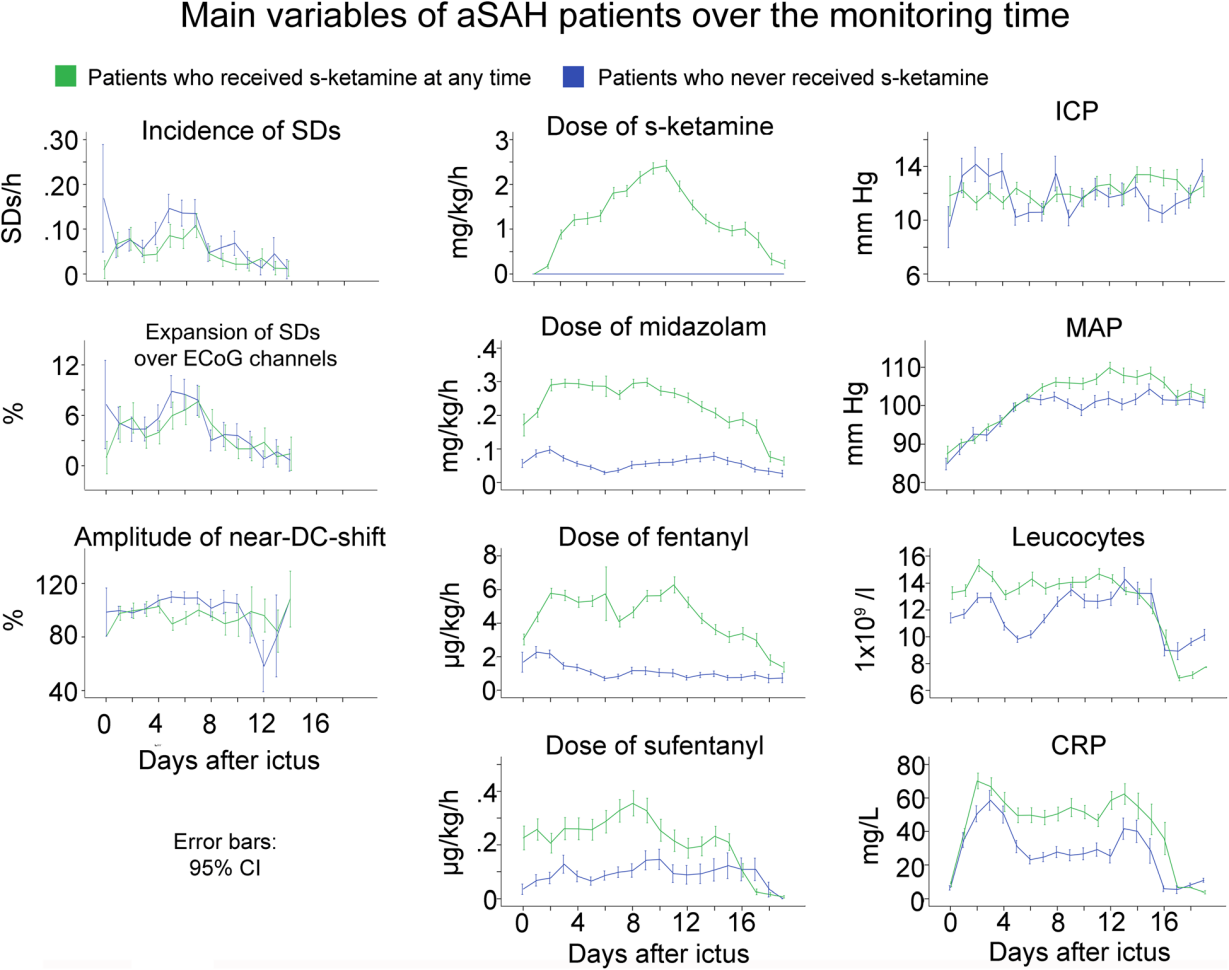
Development of multiple variables over time in patients with aSAH who received s-ketamine at any time during the monitoring versus patients who did not. Patients treated with s-ketamine also received higher doses of the other sedatives and analgesics. Accordingly, also CRP was higher which might reflect a higher rate of infections under sedation. The time courses illustrate the selection bias of the study with patients requiring s-ketamine at any time during the monitoring having more severe aSAH. The means are presented here with 95% confidence interval (CI)

The 66 patients were first divided in two groups according to whether they received s-ketamine at any time of the monitoring or not. Patients who did not receive s-ketamine had 622 SDs overall, and the average number of SDs detected per patient was 18.9 ± 29.2. Patients who received s-ketamine had a total of 422 SDs, with an average of 12.8 ± 17.6 SDs per patient. The SD frequency was 0.08 ± 0.32 SDs/h in patients who never received s-ketamine and 0.06 ± 0.25 SDs/h in patients who did receive s-ketamine. Variables comparing both groups are presented in Fig. 4.

To determine whether there was a difference in SD incidence between the two groups, we first fitted a negative binomial model using the SD count per patient as outcome variable and the group (s-ketamine given vs. not given) as covariable. No significant difference in SD counts was found (coefficient − 0.39 (− 1.10; 0.33), *p* = 0.288), most likely because 368 SDs had already occurred in the s-ketamine group before the s-ketamine administration was initiated. Because most of the hourly counts were 0, we additionally performed a logistic model. The sum per person was calculated and then dichotomized (> 0 vs. =0). Again, no difference was found between the groups (OR 0.59 (0.18; 1.91), *p* = 0.381). The expansion of SDs (percentage of channels invaded by SD in proportion to the channels available) was calculated for both groups, and no significant difference was found (coefficient 4.15 (− 7.41; 15.72), *p* = 0.482). The amplitude of the near-DC-shift was also compared using a linear mixed model, but similarly showed no difference (coefficient − 7.68 (− 26.10; 10.73), *p* = 0.414) on the patient level.

### S-ketamine effect on the incidence of SDs in hourly pooled data

In Fig. 2, an example of a patient is given in whom clustered SDs were blocked by s-ketamine followed by a recovery of the spontaneous activity. In Fig. 3, we provide an example of a patient with DCI after aSAH in which the ECoG is also shown before and during s-ketamine application (Fig. 3b, c). However, not all SDs were blocked under s-ketamine in this case, although their characteristics changed.

**Fig. 5:**
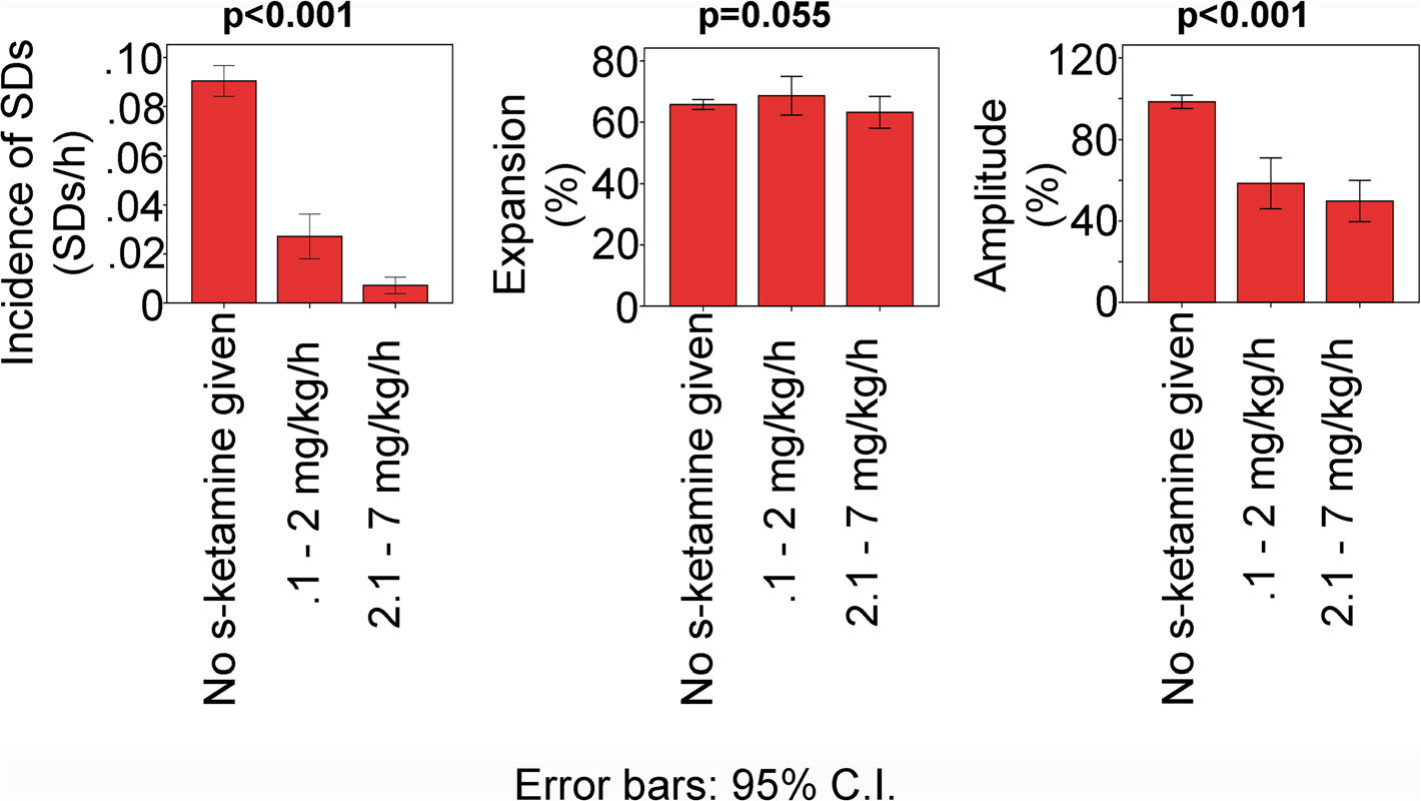
Incidence and characteristics of SDs in patients with aSAH dependent on s-ketamine. Patient data was hourly pooled according to whether s-ketamine was not given, given at a low dose or at a high dose. Data from the same patient could be in all three bars. For the statistical analysis, different models were used as described in “Results.” *p* values < 0.05 mean that there was a difference between groups with s-ketamine versus control without s-ketamine

**Fig. 6:**
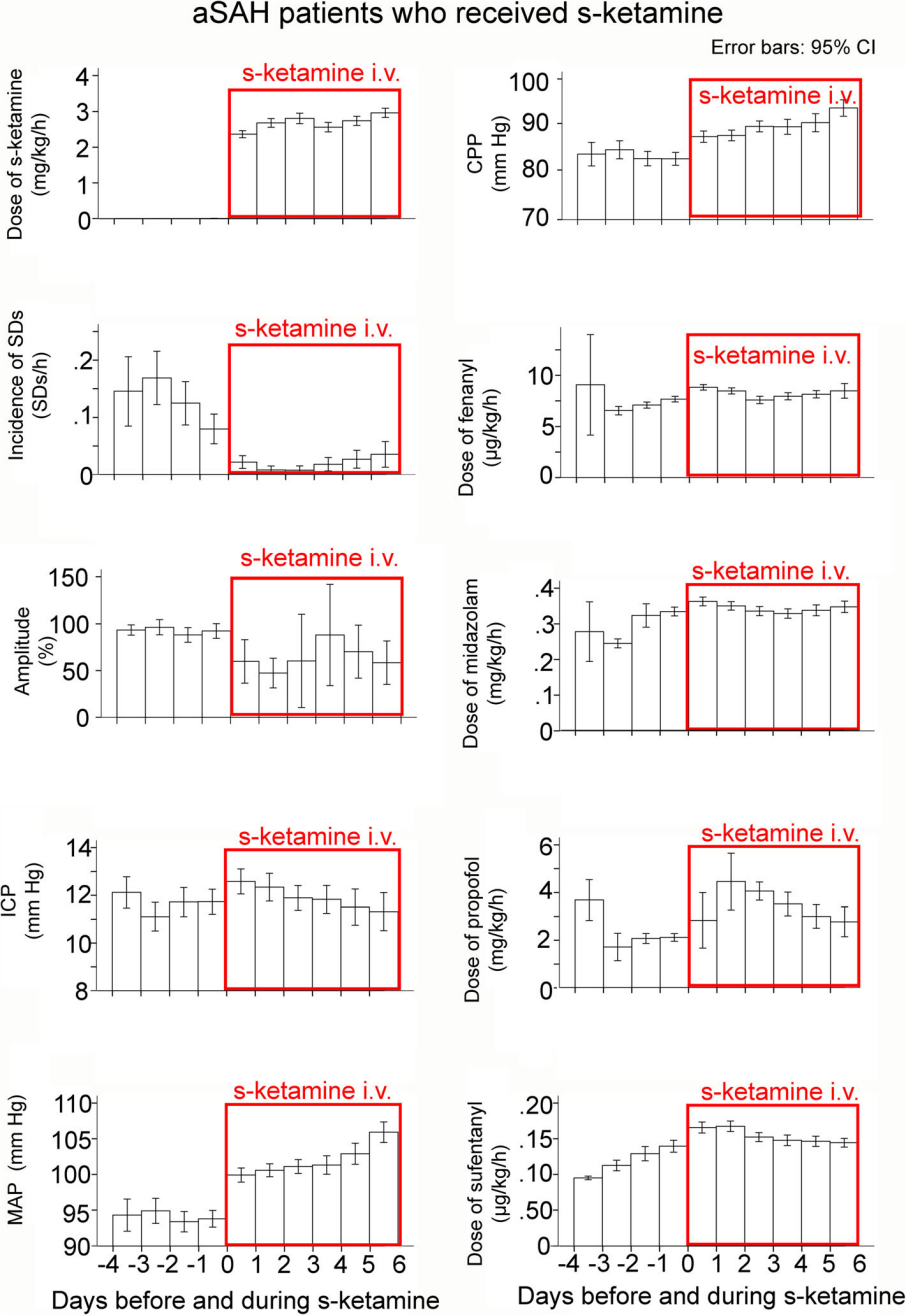
Data classified as a function of the time point when s-ketamine was applied. The means are presented here with 95% confidence interval (CI). When s-ketamine was discontinued, subsequently recorded data was not used for this analysis. Note that (i) the mean dose of s-ketamine was above 2 mg/kg BW/h and (ii) all variables analyzed showed a difference before and after s-ketamine. The incidence of SDs showed a clear and lasting reduction in response to s-ketamine

We next grouped hourly data on the basis of whether or not s-ketamine was given in that hour. All 66 patients were included in this analysis, and the main patient findings are summarized in Fig. 5. The effect of s-ketamine on SD incidence was initially analyzed using a Poisson model with a random intercept for patient. We included the MAP in the model as a controlled variable because of the higher MAP during s-ketamine treatment (Fig. 6) and because it was previously found in patients with TBI that a higher MAP is associated with lower incidence of SDs [39]. We observed a significant decrease of the incidence of SDs when s-ketamine was given (coefficient − 1.83 (− 2.17; − 1.50), *p* < 0.001). Because there were many observations with SDs = 0, a logistic regression model for the binary outcome SDs = 0 vs. SDs > 0 was estimated. The logistic regression model with random intercepts for patient showed a significant decrease in the incidence of SDs (OR 0.13 (0.08; 0.19), *p* < 0.001). Linear mixed models were used to examine (i) the percentage of channels involved in SD in proportion to the channels available (expansion in %) and (ii) the near-DC-shift amplitude. No significant change of expansion was found when s-ketamine was given (SD expansion: coefficient 6.24 (− 0.01; 12.50), *p* = 0.05). In contrast, the amplitude of the near-DC-shift significantly decreased (coefficient − 29.20 (− 46.06; − 12.35), *p* < 0.001).

Thereafter, the hourly pooled data was further divided into low-dose (0.1–2.0 mg/kg BW/h) and high-dose (2.1–7.0 mg/kg/h) segments. The low-dose range is the therapeutic range supported by safety studies according to the manufacturer (Pfizer, New York, USA) whereas the high-dose range is administered by intensivists in individual cases. The incidence of SDs was compared between low- and high-dose ranges using a Poisson model with a random intercept for patient using MAP as a controlled variable. High-dose s-ketamine was associated with further significant decrease in the incidence of SDs (coefficient − 1.10 (− 1.71; − 0.49), *p* < 0.001). Again, there were many observations with SDs = 0. Therefore, a logistic regression model for the binary outcome SDs = 0 vs. SDs > 0 was estimated. The logistic regression model with random intercepts for patients revealed a significant difference between the low- and high-dose ranges (OR 0.33 (0.17; 0.63), *p* < 0.001). However, no significant difference was found in either SD expansion (coefficient − 3.23 (− 10.64; 4.19), *p* = 0.393) or the near-DC-shift amplitude (coefficient − 2.64 (− 18.11; 23.38), *p* = 0.803) between the low- and high-dose ranges.

### Changes before and after s-ketamine application

We also examined differences between the first day when s-ketamine was given and the preceding day. Only the 33 patients who received s-ketamine were considered (Fig. 6). There was a difference in all examined variables: incidence of SDs (coefficient − 1.38 (− 0.75; − 2.00), *p* < 0.001), ICP (coefficient 1.30 (0.72; 1.88), *p* < 0.001), MAP (coefficient 6.73 (5.80; 7.66), *p* < 0.001), fentanyl (coefficient 1.47 (1.23; 1.72), *p* < 0.001), midazolam (coefficient 0.07 (0.06; 0.08), *p* < 0.001), propofol (coefficient − 0.27 (− 0.18; − 0.37), *p* < 0.001), and sufentanyl (coefficient − 0.06 (− 0.04; − 0.09), *p* < 0.001). Notably, it can be seen in Fig. 6 that the incidence of SDs gradually increased again after several days of s-ketamine, but clearly remained below the level without s-ketamine.

In addition, we determined the number of clustered SDs per recording day before and after s-ketamine administration, as described in “Materials and methods” and following the recent COSBID recommendations [37]. Whereas 1.3 ± 2.7 clustered SDs per recording day were observed before s-ketamine administration, only 0.1 ± 0.5 were observed thereafter (Wilcoxon signed rank test, *p* < 0.001). Finally, it should be mentioned that s-ketamine has a double effect on spontaneous brain activity. When administered during a cluster of SDs and the SDs are blocked, as in the example shown in Fig. 2, spontaneous brain activity can recover and the power of the activity re-increases. On the other hand, s-ketamine is assumed to have a direct suppressive effect on spontaneous brain activity, which means that the power of brain activity should decrease if no cluster takes place immediately before the administration of s-ketamine. This double effect could explain why we did not observe a significant change of the mean power of spontaneous brain activity between the day before s-ketamine administration and the first day during s-ketamine administration (Wilcoxon signed rank test, *p* = 0.096).

## Discussion

The foundations of the excitotoxicity concept in acute brain injury were first established by Anthonie van Harreveld in the 1950s. In a landmark paper, he proposed that glutamate is an important excitatory neurotransmitter, causing contraction when applied to crustacean muscle [40]. Yet, he also found that glutamate triggers the pathologic reaction of SD when applied to the cerebral cortex, and conversely, provided evidence that SD causes the release of glutamate into the interstitial space when it invades a normal, undisturbed cortex. The toxic aspect of this process was evidenced by an increased tissue impedance during SD, which suggested a shift of electrolytes from the extra- to the intracellular compartment, as reviewed recently [7]. Electron microscopy then confirmed the swelling of neuronal somas, dendrites, and mitochondria as a consequence of the breakdown of electrochemical gradients. Importantly, these changes—the release of glutamate and the cytotoxic edema—occurred similarly as a result of both SD and severe cerebral ischemia, suggesting that the former mediates damage from the latter. Unfortunately, these insights were lost when Olney (1969) [41] and then Choi (1980s) [42] were credited with the discovery of glutamate excitoxicity, which was then characterized and studied without consideration of the SD mechanism. Thus, neuroprotectants against stroke or TBI were not developed or assessed for dosing efficacy against SDs occurring in metabolically compromised regions [43]. Instead, they aimed at doses with negligible psychotropic effects, although the literature suggested that these would not be sufficient to block SDs where they are harmful. Accordingly, there was no evidence of significant benefit or harm from these drugs in the ensuing clinical trials [44].

Maintaining wakefulness and avoiding delirium are, without doubt, important goals in alert patients with stroke or TBI. For example, sedation often necessitates intubation and ventilation. Each day of mechanical ventilation increases the risk of pneumonia [45], which is in turn associated with worse outcome [46]. Thus, when aiming at the administration of neuroprotectants to alert patients, drug developers rightly wanted to avoid psychotropic side effects. These conditions may have been self-defeating, however, as neuroprotective benefit of NMDAR antagonists was likely not possible at such doses. On the other hand, these considerations suggest that NMDAR antagonists may still be promising candidate drugs to protect neurons when patients with acute cerebral injuries require sedation. In this respect, it is interesting that one of the NMDAR antagonists has found its way into routine neurocritical care practice to sedate patients with acute cerebral injuries, including aSAH. This development has occurred in a more or less unsystematic fashion, driven by clinical experience rather than higher-level scientific evidence, and concerns the open channel NMDAR blocker, ketamine.

Ketamine is a dissociative anesthetic causing dose-related analgesia and unconsciousness. Observable analgesia is achieved at doses between 0.1 and 0.5 mg/kg, while anesthesia requires doses over 1 mg/kg [47]. The active enantiomer S(+) ketamine (s-ketamine) is two times stronger than the racemic form and four times stronger than the R(−) enantiomer [48]. In contrast to the USA, where s-ketamine is not licensed and the racemic mixture is used, s-ketamine is licensed and frequently applied in Germany. The upper therapeutically recommended dose of s-ketamine for sedation is 2 mg/kg BW/h, but, if deemed necessary, neurointensivists are using doses of up to 7 mg/kg BW/h. Combination with the GABA_A_ agonist midazolam is standard to avoid ICP increases that are presumably related to ketamine’s psychotomimetic effects.

The present study is the largest one to date on the effect of s-ketamine on SDs in aSAH patients. We found that s-ketamine administration was associated with a reduced SD incidence and a reduced amplitude of the near-DC-shift. Importantly, SDs were most potently inhibited by doses of s-ketamine that are above clinically recommended doses, but are nonetheless given by neurointensivists in individual cases. An important limitation is the retrospective study design. This led to the selection bias that the sicker patients were treated with s-ketamine. Thus, patients receiving s-ketamine more frequently showed WFNS 4 or 5 values at admission and had more severe aSAH on the modified Fisher scale [36], which is the most likely reason why s-ketamine-treated patients had a worse outcome. However, it cannot be completely excluded that the s-ketamine group experienced more severe secondary complications due to, for example, stronger sedation, as explained above. This can only be clarified in a prospective trial. Such a trial should be preceded by a feasibility study, as we do not yet know enough about the practicability of neuromonitoring-guided treatment protocols in general and about the aggressiveness, timing, and duration of the treatment of SDs in particular.

Non-competitive NMDAR antagonists such as ketamine bind at the phencyclidine receptor site within the NMDAR-controlled non-selective cation channel. A problem, previously observed in rats and specific to this type of NMDAR antagonists, is the use-dependent development of tolerance, which typically appeared after several SD waves were elicited under ketamine [35]. Use-dependency refers to the fact that the tolerance only developed in presence of glutamate, the receptor agonist, which is repeatedly released at high concentrations during repetitive SDs [49, 50]. It was proposed that this results from conformational changes at the binding site [49]. In our patients, we did not find clear evidence for the development of tolerance against s-ketamine. The reservation, however, is that the group of Bures had to induce SDs in rats at regular intervals of ~ 15 min in order to induce the use-dependent tolerance. Therefore, we cannot rule out a tolerance in patients when SDs occur in a high rate cluster [4]. Nonetheless, the present results are important because they dispel concerns that s-ketamine would rapidly and in a clearly visible manner lose efficacy against SDs when applied to patients over a prolonged period. Another concern is that ketamine could increase ICP. Although significant, we found only a small and clinically irrelevant increase. MAP also increased. In the DCI phase, however, this is rather a positive side effect.

Our results do not address the other important concern that NMDAR antagonists are generally insufficient to block terminal SD in severely ischemic tissue [23–26]. Rodent brain slice experiments suggested that additional ion channels are recruited into the SD-generating mechanism with increasing metabolic failure [26, 51, 52]. Thus, only a bathing medium containing high concentrations of, e.g., DNQX/NBQX to block all AMPA/kainate receptors, the combination of MK-801 and APV to potently block NMDARs, and bicuculline methiodide to block GABA_A_ receptors succeeded in preventing SD in severely ischemic/hypoxic tissue. The required concentrations were so high that it is difficult to see how they could be achieved in vivo without causing significant toxic side effects [52]. Yet, as explained above, SD can trigger spreading ischemia even in tissue with relatively preserved metabolism [19]. This SD-mediated vasoconstriction can then induce severe energy deprivation in the tissue. Thus, by blocking the spread of SDs into such regions of impaired neurovascular coupling, NMDAR antagonists may still offer substantial neuroprotective benefit. Recent monitoring of ECoG, regional cerebral blood flow, and tissue partial pressure of oxygen in aSAH patients supported this notion [10]. For example, in one case, a short-lasting SD triggered a brief hypoperfusion at one optoelectrode pair. From there, the SD spread to the adjacent optoelectrode pair, where it caused a spreading ischemia of more than 50 min duration, leading to a so-called negative ultraslow potential (NUP). The NUP specifically refers to a negative potential component during progressive recruitment of neurons into cell death in the wake of SDs [7]. Serial neuroimages proved that an infarct developed at the recording site.

Another concern complicating the neuroprotective use of NMDAR antagonists is a potentially beneficial effect of SDs in healthy tissue, in contrast to metabolically impaired tissue. In otherwise normal tissue, SDs upregulate growth factors, stress response proteins, and inflammatory mediators, may have preconditioning actions, and could enhance plasticity and regeneration [12, 27, 53]. This remains a theoretical consideration, as there is no conclusive evidence that SDs are beneficial in regions remote from injured cortex, and the net effect of inhibiting SDs with NMDAR antagonists was protective in most experimental focal ischemia studies [54–58]. Nevertheless, a recent experimental study in brain slices should not go unmentioned in which ketamine significantly reduced the neuronal Ca^2+^ surge during SD in parallel with SD’s duration and accelerated the recovery of postsynaptic potentials after SD in tissue with mildly impaired metabolism, but SD initiation and propagation remained preserved [59]. These results were interpreted to suggest that a lower dose might be more beneficial than a higher dose of ketamine.

In the present study, the occurrence of electrographic seizures in the ECoG recordings was not systematically analyzed, which is a limitation. Another limitation is that we did not perform continuous scalp electroencephalography to detect electrographic seizures. However, in a previous pilot study of 25 patients with aSAH, we found electrographic seizures in 12% of the ECoG recordings [60]. Ketamine inhibits epileptic activity in animal models [61] and has been successfully used as a third- or fourth-line treatment of refractory status epilepticus in patients [62]. On this basis, it is expected that the administration of ketamine should not only inhibit SDs but also electrographic seizures. This could be another argument for the use of ketamine and should be systematically studied in a prospective trial. In our previous study, we also observed that patients who developed late epilepsy within 3 years after the hemorrhage showed a higher peak number of SDs during the early clinical course [60]. In the present study, we did not investigate whether inhibition of SDs by s-ketamine reduced the frequency of late epilepsy, but we recommend that this interesting research and clinical question should be included in a future prospective trial.

Another limitation is the use of a single electrode strip to detect SDs. Although SDs propagate widely from metabolically stressed zones, there is evidence from both clinical studies, including the present one, and animal studies that a fraction of SDs is rather localized [33, 63–66]. Therefore, if neuromonitoring is used to guide the administration of ketamine, clusters of SDs may be overlooked if the electrode strip is too far away from the metabolically disturbed region. No significant complications have been observed using a single electrode strip to detect SDs [45]. The strip does not require an operation for the removal and can be easily withdrawn at the bedside. Thus, the current practice is a trade-off between patient safety and benefit. However, by using an additional strip, the significance of the recordings could presumably be increased without significantly increasing the risk for the patient. Alternatively, it was proposed to place depth electrodes subdurally, which could facilitate implantation through a burr hole and would allow more electrodes to be implanted that could still be removed at the bedside [9]. The advantage of placing depth electrodes in the subdural space and not in the parenchyma would be that injury and inflammation of the parenchyma would be avoided [37]. At the same time, the probability of detecting SDs would increase as they are recorded from more than one cortical location [67].

## Conclusions

ECoG neuromonitoring offers the potential advantage of performing early treatment stratification according to SDs detected in real time, and then to document the response to a neuroprotective intervention with subsequent dose adjustments in an iterative fashion [27]. Furthermore, SDs are a promising diagnostic summary measure because they propagate widely from metabolically stressed zones, thereby affording even remote detection of newly developing injury. A pressing clinical question in this context is whether sedation-requiring individuals undergoing clusters of SDs in the wake of acute cerebral injuries should receive the combination of midazolam and ketamine rather than midazolam and propofol—the usual first-line combination treatment in neurocritical care—in order to prevent SDs that carry the risk of triggering spreading ischemias. The present results provide the foundation to address this question with a feasibility study for a neuromonitoring-guided, randomized, blinded, multicenter, proof-of-concept trial. The fundamental question is whether ketamine should be started earlier in the event of SDs, in contrast to the delay in ketamine use observed here in current practice, in order to offer a greater chance for neuroprotection. In such a trial, ketamine could be tested at both the currently recommended dose for sedation and a higher one that is still often administered in current clinical practice.

## Data Availability

Electronic recording, processing, and storage of the data were approved by the data protection officer of the Charité – Universitätsmedizin Berlin. The raw datasets analyzed during the current study are not publicly available because the patient’s informed consent only permits the data analysis and publication by the investigators.

## Abbreviations

ACA: Anterior cerebral artery
aSAH: Aneurysmal subarachnoid hemorrhage
BW: Body weight
CI: Confidence intervals
CPP: Cerebral perfusion pressure
CRP: C-reactive protein
CT: Computed tomography
DC: Direct current
DCI: Delayed cerebral ischemia
ECoG: Electrocorticography
eGOS: Extended Glasgow Outcome Scale
ICP: Intracranial pressure
MAP: Mean arterial blood pressure
MCA: Middle cerebral artery
MTT: Mean transit time
NMDAR: *N*-Methyl-d-aspartate receptor
NUP: Negative ultraslow potential
SD: Spreading depolarization
TBI: Traumatic brain injury
WFNS: World Federation of Neurosurgical Societies scale
